# Molecularly Imprinted Magnetic Fluorescent Nanocomposite-Based Sensor for Selective Detection of Lysozyme

**DOI:** 10.3390/nano11061575

**Published:** 2021-06-15

**Authors:** Xin Zhang, Bo Tang, Yansong Li, Chengbin Liu, Pengfei Jiao, Yuping Wei

**Affiliations:** 1 School of Life Science and Agricultural Engineering, Nanyang Normal University, Nanyang 473061, China; li.yansong1989@163.com (Y.L.); liuchengbin@tio.org.cn (C.L.); dongbx@nynu.edu.cn (P.J.); ypwei@ipe.ac.cn (Y.W.); 2 Key Laboratory of Molecular Medicine and Biotherapy, School of Life Science, Beijing Institute of Technology, Beijing 100081, China; 3120170650@bit.edu.cn

**Keywords:** molecular imprinting, magnetic nanoparticles, quantum dots, fluorescent sensor

## Abstract

A new strategy for the design and construction of molecularly imprinted magnetic fluorescent nanocomposite-based-sensor is proposed. This multifunctional nanocomposite exhibits the necessary optics, magnetism and biocompatibility for use in the selective fluorescence detection of lysozyme. The magnetic fluorescent nanocomposites are prepared by combining carboxyl- functionalized Fe_3_O_4_ magnetic nanoparticles with *l*-cysteine-modified zinc sulfide quantum dots (MNP/QDs). Surface molecular imprinting technology was employed to coat the lysozyme molecularly imprinted polymer (MIP) layer on the MNP/QDs to form a core-shell structure. The molecularly imprinted MNP/QDs (MNP/QD@MIPs) can rapidly separate the target protein and then use fluorescence sensing to detect the protein; this reduces the background interference, and the selectivity and sensitivity of the detection are improved. The molecularly imprinted MNP/QDs sensor presented good linearity over a lysozyme concentration range from 0.2 to 2.0 μM and a detection limit of 4.53 × 10^−3^ μM for lysozyme. The imprinting factor of the MNP/QD@MIPs was 4.12, and the selectivity coefficient ranged from 3.19 to 3.85. Furthermore, the MNP/QD@MIPs sensor was applied to detect of lysozyme in human urine and egg white samples with recoveries of 95.40–103.33%. Experimental results showed that the prepared MNP/QD@MIPs has potential for selective magnetic separation and fluorescence sensing of target proteins in biological samples.

## 1. Introduction

Mn^2+^-doped zinc sulfide quantum dots (Mn^2+^: ZnS QDs) exhibit low toxicity and are synthesized by using Mn doped with non-heavy metal elements [1]. As a fluorescent nanomaterial, Mn^2+^: ZnS QDs have unique optical properties, such as symmetric emission, size-dependent emission-wavelength tunability, photochemical stability, and low biotoxicity [2,3,4,5]. These features of Mn^2+^: ZnS QDs often result in their use as fluorescent nanosensors with improved sensitivities for the detection of biomacromolecules [6,7]. Fe_3_O_4_ magnetic nanoparticles (MNPs) are an important class of magnetic materials and have attracted considerable attention in recent years because of their unique advantages, such as physicochemically tailored surface properties, facile synthesis, chemical stability and operational stability [8,9]. Fe_3_O_4_ MNPs can automate and simplify the assay process using magnetic separation, which not only facilitates sample pretreatment and purification, but also improves the efficiency of the assay process [10,11]. Thus, Fe_3_O_4_ MNPs are widely used in biochemical separations, sensor applications and bioanalyses [12,13,14,15].

By combining Fe_3_O_4_ MNPs and Mn^2+^: ZnS QDs, an advanced magnetic fluorescent nanocomposite can be prepared for the detection of biomacromolecule [16]. Fe_3_O_4_ MNPs involving Mn^2+^: ZnS QDs can facilitate magnetic separation and a quick fluorescent response due to fast electron transfer rates [17,18]. These prepared MNP/QDs can serve as fluorescence biological probes or biosensing agents and can directly tag and separate biomolecules in a process driven by an external magnetic field. Furthermore, this MNP/QDs sensor integrates the magnetic separation process with the optical detection process, greatly facilitates the analysis and detection process and is expected to improve detection efficiency and sensitivity [19,20,21,22]. However, it is still difficult to efficiently and specifically recognize target analytes in complex systems. The application of MNP/QDs to the separation and detection of target analytes is hindered by nonspecific adsorption and interference from other components, which seriously affects the accuracy and restricts the specificity and sensitivity of the detection.

Based on this background, molecular imprinting technology was employed herein to tailor the sensitivity of MNP/QDs-based-sensor. Molecularly imprinted polymers (MIPs) have been extensively explored for the detection of various target molecules by their attractive advantages, such as chemical stability, high affinity and predetermined selectivity for target molecules [23,24,25,26,27,28]. However, protein imprinting still poses a challenge because of the conformational flexibility, large molecular size, and solubility properties of proteins [29,30]. Designing biosensors based on surface imprinting and the use of nanocomposites is a promising strategy for addressing these difficulties [31,32]. Artificial receptors with molecular recognition ability can be constructed by coating molecularly imprinted polymers on the surfaces of MNP/QDs nanocomposites to form a “core-shell” structure, and the physical and chemical properties of the polymers are retained. MNP/QD@MIPs introduce molecular recognition units to MNP/QD nanocomposites, which can improve the binding kinetics, binding capacity, specificity, and fluorescence efficiency [33,34,35]. Moreover, the MNP/QD@MIPs-based sensor combines the magnetic properties and optical readout characteristics of MNP and QDs, as well as highly selective of molecularly imprinted technique in one composite system, which can easily, quickly and selectively recognize target molecules [36,37].

In the present work, lysozyme (Lyz) was used as the template protein. Lyz is an important biomarker in clinical diagnosis, increasing levels of Lyz are potential indicators for leukemia and renal diseases [38,39]. Thus, the selective and sensitive detection of Lyz is of considerable importance. To date, a variety of analytical technologies have been developed for the detection of Lyz, such as enzyme-linked immune sorbent assay (ELISA), high performance liquid chromatography (HPLC), mass spectrometry, electrochemistry, and biosensors [40,41,42,43,44]. Although these methods possess sensitivity and accuracy, many of them are limited by time-consuming pretreatments, instrument dependence, and low selectivity. Therefore, the current work presents a strategy for the specific recognition of Lyz through the fabrication of a molecularly imprinted magnetic fluorescent nanocomposite-based sensor. The sensor integrates the recognition ability originating from an imprinted cavity and the magnetic separation and fluorescence detection processes. Compared to traditional detection methods, the MNP/QD@MIPs-based sensor can be used directly for the magnetic separation of the lysozyme without complex equipment or any further pretreatment procedures, such as centrifuge or filtration, and then for the fluorescence determination of Lyz with high selectivity. In addition, this method does not involve any other pretreatment procedures or additional instruments. The MNP/QD@MIPs-based sensor shows a rapid magnetic response and a quick fluorescence determination process. In this study, the *l*-cysteine-modified ZnS QDs were combined with carboxyl-functionalized Fe_3_O_4_ MNPs and were adopted as cores. Acrylamide and *N*-isopropylacrylamide were used together as functional monomers to ensure the bioactivities of proteins. The protein imprinting layer was immobilized on the MNP/QDs composites to form a “core-shell” structure. This MIP layer not only provides selectivity for the template protein but also prevents interfering molecules from combining with the MNP/QDs. The molecularly imprinted MNP/QDs are used as a highly selective and effective Lyz magnetic fluorescence sensor. This prepared MNP/QD@MIPs sensor is accurate and selective in the direct detection of Lyz without the need for any time-consuming procedures, and can easily be reused and recycled; therefore, it shows promise in biosensing applications.

## 2. Materials and Methods

### 2.1. Materials and Reagents

ZnSO_4_∙7H_2_O, Na_2_S∙9H_2_O, MnCl_2_∙4H_2_O, FeCl_3_∙6H_2_O and Na_3_Cit∙2H_2_O were obtained from Beijing Chemical Works (Beijing, China). Acrylamide (AAM), *N,N′*-methylenediacrylamide (MBAAM), *N*-isopropylacrylamide (NIPAAm), sodium dodecyl sulfate (SDS), *N,N,N′,N′*-tetramethylethylethylenediamine (TEMED) were pruchased from Sinopharm Chemical Reagent Co. Ltd. (Beijing, China). *L*-cysteine, 1-ethyl-3-(3-dimethylaminopropyl) carbodiimide (EDC), and N-hydroxysuccinimide (NHS) were received from Macklin Biochemical Co. Ltd. (Shanghai, China). Cytochrome C (Cyt, MW12.4 kDa, pI 10.2), Lyz (Lyz, 14.4 kDa, pI 11.2), bovine serum albumin (BSA, MW 67 kDa, pI 4.9), and ovalbumin (OVA, 45 kDa, pI 4.7) were purchased from Sigma-Aldrich Co. (Shanghai, China).

### 2.2. Characterizations

The fluorescence spectra were determined by FL-970 spectrofluorometer (Techcomp, Shanghai, China). The magnetic properties were analyzed with a 7307 vibrating sample magnetometer (VSM, Lake Shore, MD, USA). Transmission electron microscopy (TEM) micrographs of magnetic nanomaterials were performed on an H-800 transmission electronic microscope (Hitachi, Tokyo, Japan). X-ray diffraction (XRD) was carried out using an Ultima IV diffractometer (Rigaku, Tokyo, Japan). The composition of the nanomaterials was analyzed using a Sigma 500 field emission scanning electron microscope (FESEM, ZEISS, Jena, Germany) coupled with energy dispersive X-ray (EDX) spectroscope. UV/vis adsorption spectra were measured with a Specord 210 plus spectrophotometer (Jena, Germany).

### 2.3. Synthesis of MNP/QD

Carboxyl-modified Fe_3_O_4_ (Fe_3_O_4_@COOH) MNPs and *l*-cysteine-modified QDs were prepared according reported methods with some modifications [9,42]. *l*-cysteine was coated on the surface of the Mn^2+^: ZnS QDs through ligand competition. Fe_3_O_4_@COOH magnetic nanoparticles were synthesized in one step by a facile solvothermal method. For the details of the synthesis process, see the Supplementary Materials.

The Fe_3_O_4_ MNPs and *l*-cysteine-modified Mn^2+^: ZnS QDs were combined via the EDC/NHS process. In brief, 100 mg Fe_3_O_4_ MNPs was ultrasonically dispersion in 100 mL citrate buffer solution (0.02 mol/L, pH = 6.4) to prepare magnetic fluids. The magnetic fluids were injected into 200 mL EDC/NHS activating agent and stirred for 30 min. Thereafter, 120 mg *l*-cysteine-modified ZnS QDs were added to the mixture, which was subjected to ultrasonication for 40 min. Finally, the resulting solution was kept stirring at 30 °C under N_2_ for 20 h. The products were collected by magnet, and washed to remove the residual reactants.

### 2.4. Fabrication of MNP/QD@MIPs Nanocomposites-Based Sensor

**Figure 1:**
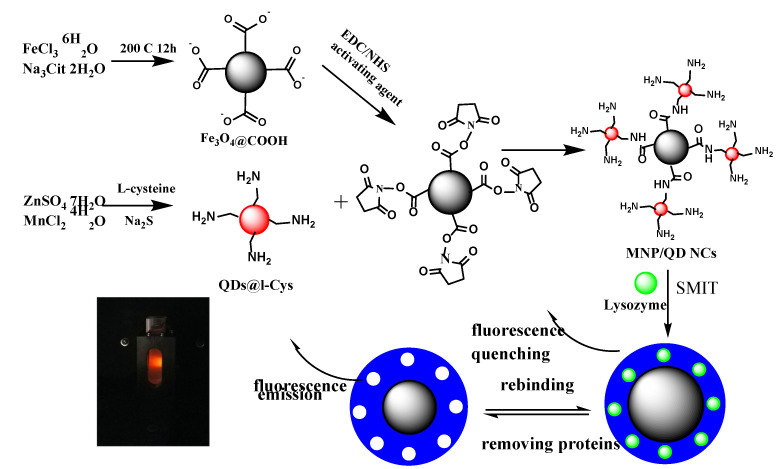
Schematic of the synthesis of MNP/QD@MIP-based sensor. SMIT: surface molecularly imprinting technology.

Figure 1 illustrates the strategy for synthesizing MNP/QD@MIPs. First, 20 mg Lyz, 200 mg AAM, 50 mg NIPAAm, and 250 mg MNP/QDs were dissolved in 50 mL phosphate buffer (10 mM, pH = 6.2). Second, the mixture was stirred for 1 h under N_2_ to allow full self-assembly to form a “prepolymerization” complex between the substrate, functional monomers and template protein. Then, 50 mg MBA (cross-linker) and 15 μL TEMED were added into the mixture sequentially. Subsequently, the aforementioned solution was deoxygenated and left topolymerize for 20 h in the dark. The MNP/QDs were used as substrates, and a prepolymerization complex was coated of MNP/QDs through a surface imprinting strategy. The resultant products were magnetically decanted and eluted by a mixture of SDS (1.0%) and acetic acid (10%) to remove the Lyz until no Lyz molecules were detected in the supernatants. Non-imprinted polymers (NIPs) were prepared using the same procedure but without Lyz.

### 2.5. Selectivity Experiments

The binding selectivity experiments with MNP/QD@MIPs were performed using OVA, BSA and Cyt C as comparable proteins. The protein concentration ranged from 0.2 to 2.0 μM. The MNP/QD@MIPs and MNP/QD@NIPs were magnetically separated and redispersed in the same volume of phosphate buffer solution (10 mM, pH 6.2), and then the final fluorescence intensities of MNP/QD@MIPs was measured. The imprinting factor (IF), which is the ratio of the fluorescence quenching efficiencies (F_0_/F−1) for MNP/QD@MIPs and MNP/QD@NIPs with the template protein, was used to evaluate the selective of MNP/QD@MIPs-based sensor.

Competitive binding tests were performed to further investigate the recognition properties of MNP/QD@MIPs. Binary and ternary competitive adsorption experiments were conducted using the protein mixture solution. Binary competitive experiments were performed using BSA as a competitor in a protein mixture solution, and the ternary protein solutions contained BSA, Cyt C and OVA. The concentration of Lyz was fixed at 1.0 μM and it was mixed with a gradient concentration of competitive proteins. After incubation at 25 °C for 12 min, the fluorescence intensity of the MNP/QD@MIPs was recorded. The selectivity coefficient (SC) was used to estimate the selectivity of MNP/QD@MIPs for template proteins [31]. SC is the ratio of the fluorescence quenching efficiencies (F_0_/F−1) of MNP/QD@MIPs for Lyz and competitive proteins.

### 2.6. Application in Real Samples

To order to evaluate the practicability of the MNP/QD@MIP-based sensor, MNP/QD@MIPs was used to specifically detect Lyz in human urine and egg white samples. Egg whites were separated from the yolks in fresh eggs, and the urine samples were collected from healthy volunteers. Both the samples were diluted with phosphate buffer, and the supernatant solution was collected after centrifuged at 8000 rpm for 15 min. The prepared samples were spiked with different concentrations of Lyz standards. The detection details used with the MNP/QD@MIPs is shown in the SI.

## 3. Results and Discussion

### 3.1. Characterization

**Figure 2:**
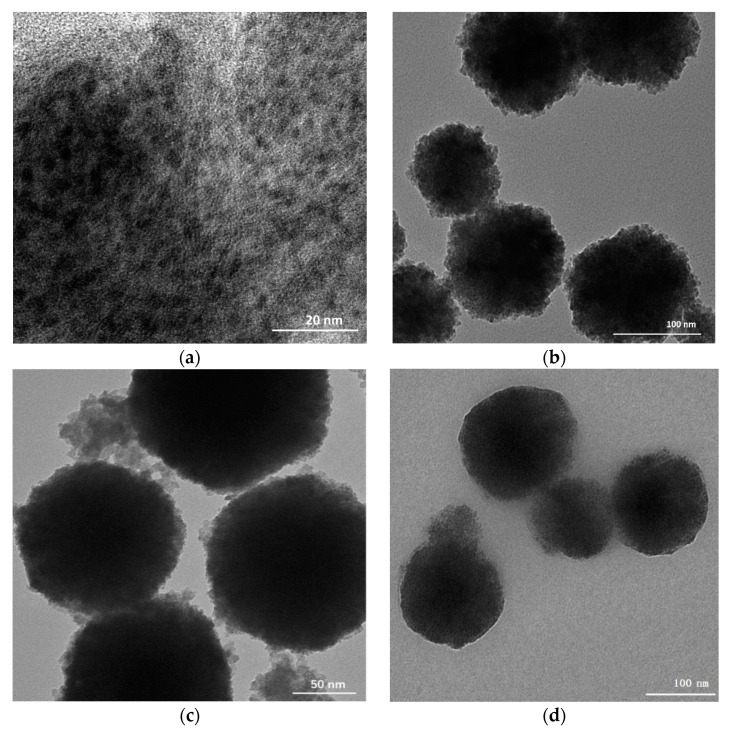
TEM images of (**a**) Mn^2+^: ZnS QDs, (**b**) MNP, (**c**) MNP/QDs and (**d**) MNP/QD@MIPs.

The structure and morphology of prepared nanomaterials were characterized by TEM and XRD. The particle size distribution of Mn^2+^: ZnS QDs, MNP, MNP/QDs and MNP/QD@MIPs were shown in Figure S1. As shown in Figure 2a,b, the mean diameters of the prepared Mn^2+^: ZnS QDs and MNPs were approximately 3.52 ± 0.30 nm and 127.93 ± 5.90 nm, respectively. Figure 2c shows that the formed MNP@QDs possessed a spherical morphology and a rough surface, and the mean diameter was approximately 133.03 ± 10.80 nm. As shown in Figure 2d, the TEM image shows that the MNP/QD@MIPs have a spherical and smoother interface, the mean diameter of the particles was approximately 145.16 ± 14.33 nm. The MNP/QD@MIPs has a smoother interface and a larger particle size because of the molecularly imprinted polymers capped on the surface of MNP@QDs by SMIT process.

The XRD pattern of MNP/QD NCs (Figure S2) showed typically characteristic diffraction peaks at 2*θ* values of 30.0°, 35.64°, 43.10°, 57.10°, and 62.82°, corresponding to the (220), (311), (400), (440), (511) planes in the standard XRD data for Fe_3_O_4_ (JCPDS No. 19-0629). Moreover, the characteristic diffraction peaks at 2*θ* values of 28.80°, 48.80°, and 56.10° for MNP/QD NCs corresponded to the (111), (220), and (311) planes of the cubic ZnS (JCPDS No. 12-0688). The XRD pattern indicates the successful formation of Fe_3_O_4_@ZnS by the combination of Mn^2+^: ZnS QDs and Fe_3_O_4_ MNPs. As shown in Figure S3, a clear contrast difference is observed between Mn2+: ZnS QDs and MNP/QDs in the EDX images, indicating that an overlay of Zn element and Fe element, further confirming the MNP/QDs structure.

**Figure 3:**
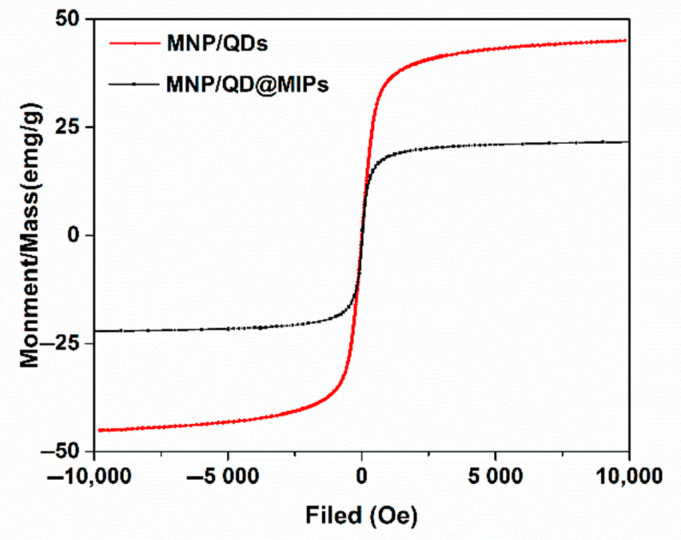
The magnetic hysteresis loops of MNP/QDs and MNP/QD@MIPs.

A VSM was used to test the magnetic properties of MNPs (Figure S4), MNP/QDs and MNP/QD@MIPs. As shown in Figure 3, both of the hysteresis loops of these prepared nanocomposites displayed typical superparamagnetic properties, and the magnetization saturation (Ms) values of MNP/QDs and MNP/QD@MIPs were approximately 45.09 and 21.88 emu/g, respectively. The decrease in Ms was caused by the MIP layers on the surface of MNP/QD@MIPs. Moreover, the superparamagnetism of the prepared MNP/QD@MIPs causes them to exhibit a rapid magnetic response under a magnetic field and good redispersion. Therefore, the prepared MNP/QD@MIPs were able to magnetically separate the target molecule rapidly and without any time-consuming procedures, enhancing the sensitivity and accuracy of fluorescence detection.

**Figure 4:**
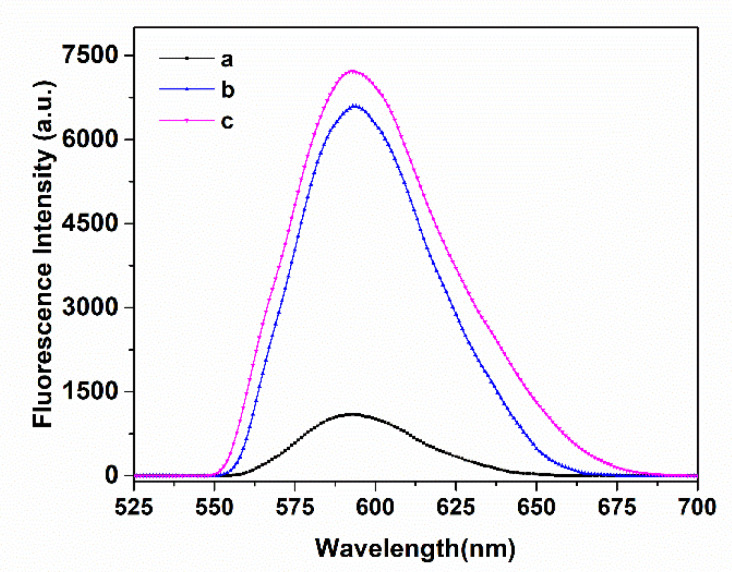
The fluorescence spectra of (a-MNP/QD@MIPs before removing lysozyme, b- MNP/QD@MIPs after removing lysozyme, and c-MNP/QDs).

The optical properties of Mn^2+^: ZnS QDs, MNP/QDs and MNP/QD@MIPs were investigated by fluorescence spectroscopy and the UV-Vis spectra. As shown in Figure S5, the Fe_3_O_4_ MNP displayed broad and strong absorption [45]. The MNP/QDs and MNP/QD@MIPs displayed a semblable absorption spectrum because of the combination of Mn^2+^: ZnS QDs, indicating that the MNPs had successfully combined with the QDs. Compared with MNP/QDs, the absorption peak of MNP/QD@MIPs was less pronounced due to the coating of Lyz MIP layer on the MNP/QDs. As shown in Figure 4, the MNP/QD@MIPs and MNP/QDs have comparable fluorescence spectra to that of Mn^2+^: ZnS QDs (Figure S6). The intensity of fluorescence peak of MNP/QD@MIPs was significantly lower before removal of the template protein because of the Lyz bound to the MIP layer of MNP/QDs. After eluting, the fluorescence intensity of MNP/QD@MIPs was dramatically restored, and the peak was less pronounced than that of MNP/QDs. Experimental results indicated that the MNP/QD@MIPs successfully retained the optical properties of MNP/QDs, which have narrow and symmetrical emission peaks and large Stoke shifts, and are suitable as substrate materials for fluorescence sensors.

### 3.2. Rebinding Experiments

#### 3.2.1. Binding Kinetics of MNP/QD@MIPs

The adsorption kinetics for Lyz with MNP/QD@MIPs and MNP/QD@NIPs are presented in Figure S7. As shown in Figure S7, the amount of binding Lyz with MNP/QD@MIPs increased significantly in the first 5 min, and then the increases in binding levelled out. After being incubation for 12 min, the adsorption of MNP/QD@MIPs for Lyz reached adsorption equilibrium. As for the control, the binding level of MNP/QD@NIPs was less than those of MNP/QD@MIPs. This is because the MNP/QD@NIPs had no imprinted sites formed during the imprinting process. The binding behavior of MNP/QD@NIPs corresponded to nonspecific binding and was unordered.

#### 3.2.2. Adsorption Isotherm of MNP/QD@MIPs

**Figure 5:**
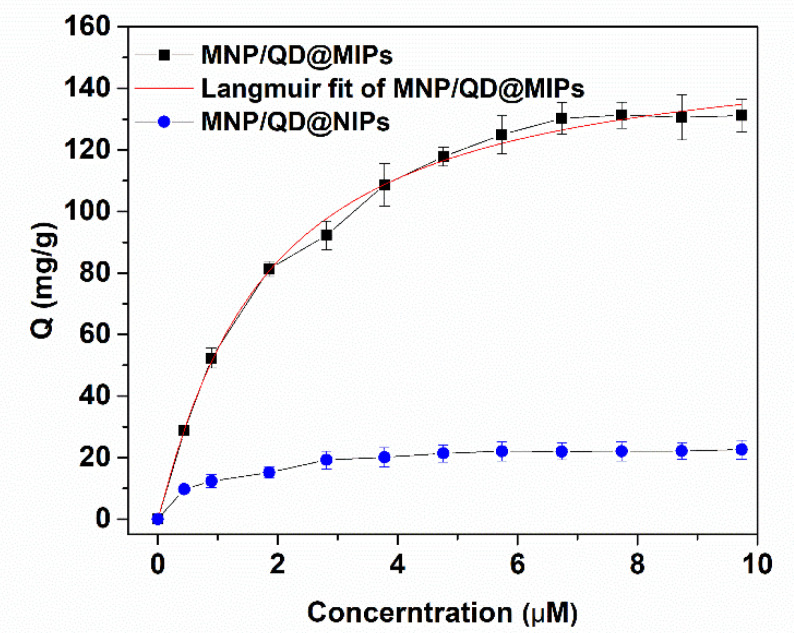
Binding isotherm and Langmuir fit of MNP/QD@MIPs and MNP/QD@NIPs.

Batch rebinding experiments for Lyz and MNP/QD@MIPs were conducted to investigate the adsorption isotherm. As shown in Figure 5, the rebinding amount of MNP/QD@MIPs was significantly higher than that of MNP/QD@NIPs, indicating that the 3D-imprinted binding site on the MIPs presented better site accessibility and more rapid mass transfer for the template protein (Lyz molecule). As the concentration of Lyz increased, the adsorption equilibrium capacity of MNP/QD@MIPs increased significantly, and the maximum amount of Lyz adsorbed at equilibrium was 127.88 mg/g. The experimental adsorption equilibrium data were fitted with the Langmuir isotherm model, and the results are shown in Figure 5. The fitting results showed that the adsorption process of MNP@MIPs for Lyz was consistent in line with the Langmuir adsorption model, and the correlation coefficient was R = 0.9925. This result indicated that the adsorption behavior of MNP/QD@MIPs and the template molecule Lyz exhibited monolayer adsorption behavior.

### 3.3. Selectivity of MNP/QD@MIPs

**Figure 6:**
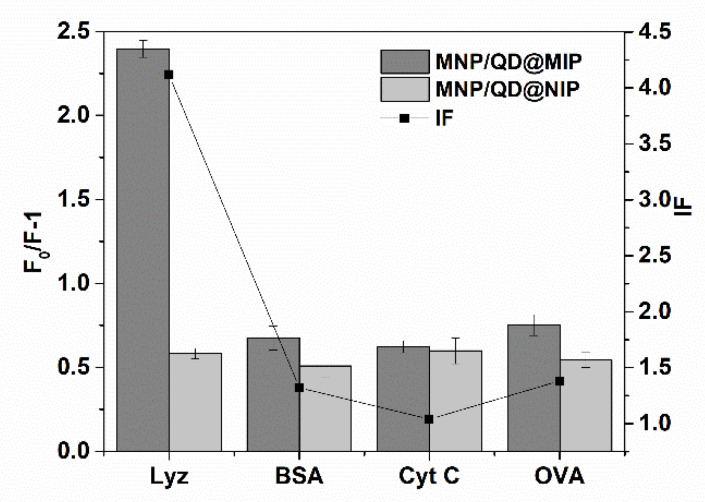
The fluorescence quenching efficiency (F_0_/F−1) and the imprinting factor (IF) of MNP/QD@MIP sensor for lysozyme, BSA, Cyt c and OVA.

Selectivity adsorption tests were performed to evaluate the selective ability of MNP/QD@MIPs for Lyz. In the selectivity experiments, BSA, Cyt C, and OVA were used as reference proteins. As presented in Figure 6, the fluorescence quenching efficiency (F_0_/F−1) of MNP/QD@MIPs with Lyz was much higher than those with other reference proteins. The imprinting factor of MNP/QD@MIPs for Lyz was 4.12, suggesting that the employed of surface imprinting process was effectively improved the selectivity and sensitivity of the detection of Lyz. Moreover, the MNP/QD@MIPs exhibited a high selectivity coefficient (SC), ranging from 3.19 to 3.85, indicating that the MNP/QD@MIP-based sensor is able to selectively recognize and detect the target Lyz in complex biosamples. In the control experiment, the amounts of Lyz and other proteins adsorbed by MNP/QD@NIPs were similar to and lower than those for MNP/QD@MIPs. This is because the template Lyz molecule is able to easily access the complementary binding site of the MNP/QD@MIPs, while there are no recognition cavities of MNP/QD@NIPs. The Lyz and other proteins were nonspecifically bound onto the MNP/QD@NIPs. The high adsorption ability of the MNP/QD@MIPs for Lyz was mainly caused by the complementary imprinting sites of the MIP layer of MNP/QDs. The selective binding of the imprinting site involves two aspects: (1) multiple weak interactions provided by the monomer, cross-linker, and Lyz molecule; and (2) the size, shape, and functional group complementarity between the imprinted cavities and template molecule in the imprinting process. These results showed that these new MNP/QD@MIPs have the ability to act as a multifunctional biosensor for direct magnetic separation and it could transform molecular recognition events into fluorescence signals for selective recognition of the target protein molecule.

**Figure 7:**
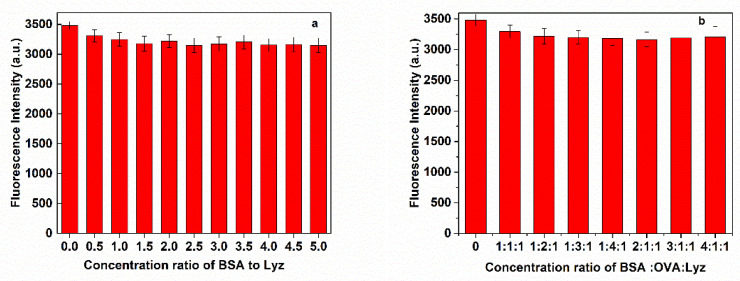
Competitive binding of MNP/QD@MIPs sensor with increasing concentration of (**a**) BSA and (**b**) BSA, OVA at a fixed concentration of Lyz (1.0 μM).

To further investigate the binding specificity of MNP/QD@MIPs for Lyz, the competition binding tests were carried out. In the binary competition adsorption experiments, the concentration of Lyz was fixed at 1.0 μM. As shown in Figure 7a, with an increasing concentration ratio of Lyz to BSA, the fluorescence of MNP/QD@MIPs was only slightly changed. The results showed that the imprinted binding sites of MNP/QD@MIPs were able to successfully and specifically recognize the target protein in a mixture of Lyz and BSA. The ternary protein mixture was also carried out to further confirm the selectivity of MNP/QD@MIPs for Lyz. BSA and OVA were chosen as competitors with the same concentrations in complex samples. As shown in Figure 7b, as the concentrations of BSA and OVA gradually increased, the fluorescence intensity change of the MNP/QD@MIPs was slightly affected by the increase in the ratio of C_lyz_: C_OVA_: C_BSA_. These results suggest that the MNP/QD@MIP-based sensor has good specific recognition ability and can be applied to selectively recognize and capture Lyz from a mixture of proteins.

### 3.4. Fluorescence Sensing of Lyz Using MNP/QD@MIPs

**Figure 8:**
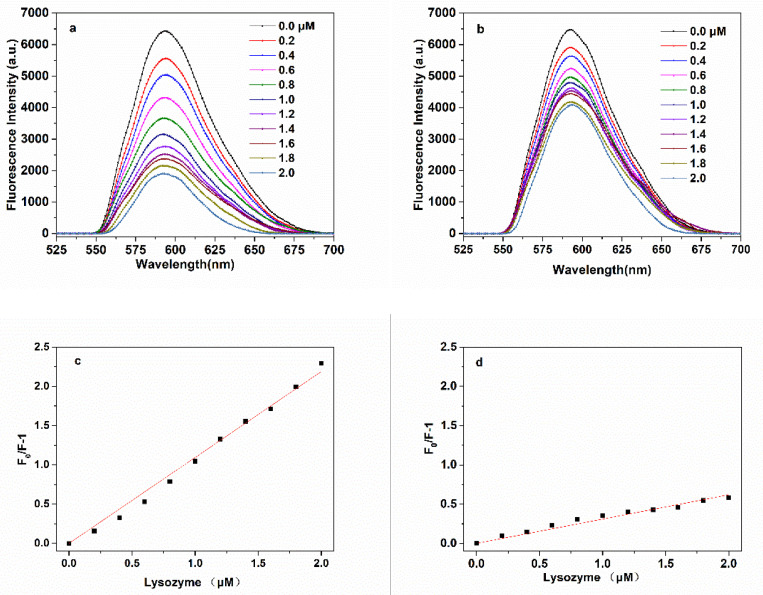
Fluorescence emission spectra of (**a**) MNP/QD@MIPs and (**b**) MNP/QD@NIPs with increasing concentrations of lysozyme; the linear calibration of the fluorescence quenching efficiency (F_0_/F−1) versus lysozyme concentration of (**c**) MNP/QD@MIPs and (**d**) MNP/QD@NIPs.

The detection mechanism is based on the specific binding of the target protein onto the imprinted cavities of the MNP/QD@MIPs, which leads to the transfer of electrons from the conduction bands of the MNP/QDs to the protein molecules [46,47]. The typical fluorescence quenching emission spectrum of MNP/QD@MIPs with Lyz is shown in Figure 8. The fluorescence quenching responses of MNP/QD@MIPs were noticeable with the increasing concentrations of Lyz. The fluorescence quenching of MNP/QD@MIPs by Lyz was much stronger than that of the NIP ones, since the lack of specific binding cavities on the MNP/QD@NIPs meant that only some Lyz molecules were bound. In contrast, the target protein Lyz absorbed onto the MNP/QD@MIPs by means of the molecular imprinting process. In addition, the extent of fluorescence quenching of the MNP/QD@MIPs was proportional to the concentration of Lyz. Thus, the MNP/QD@MIPs was used as biosensors for the fluorescence sensing of Lyz. The fluorescence quenching efficiency of MNP/QD@MIPs showed a good linear relationship with the concentration of Lyz in a range from 0.2 to 2.0 μM. The correlation coefficient for the plot of fluorescence quenching efficiency versus Lyz concentration was 0.9936 (n = 11) in the Lyz concentration range of 0.2 to 2.0 μM. The corresponding limit of detection (LOD) was calculated as 4.53 × 10^−3^ μM following the IUPAC criterion (3σ/S). The imprinting factor of MNP/@QD@MIPs for Lyz was 4.12, indicating that the imprinting process provide a significant specific recognition ability for the MNP@MIPs sensor.

### 3.5. Applications

As shown in Table 1, the MNP/QDs were used for the detection of lysozyme in egg white and human urine samples. The recoveries for the MNP/QD@MIPs detection of Lyz were 95.40–103.3%, and the RSDs were between 3.15 and 5.29 (*N* = 3). It is indicated that the developed MNP/QDs-based sensor is able to effectively and accurately determine lysozyme in real samples. The key performance of the MNP/QD@MIPs with Lyz was compared with those of different analytical techniques, and the results are shown in Table 2. As shown in Table 2, the proposed MNP/QD@MIPs-based sensor is not only convenient for magnetic separation of the target protein without any further pretreatment procedures, but also shows a rapid response. Additionally, it shows a linear range for Lyz that is comparable to or lower than those of previous works, and a lower limit of detection can be achieved.

### 3.6. Stability and Recyclability

Stability and recyclability are important properties for the application of MNP/QDs. Desorption and regeneration cycle tests were carried out to investigate the recyclability of the MNP/QD@MIPs. As shown in Figure S8, the fluorescence properties of the MNP/QD@MIPs-based sensor only decreased 8.80% after six regeneration cycles. Figure S8 shows the stability of the MNP/QD@MIPs stored in the dark conditions. The fluorescence intensity only slightly decreased after storage for 30 days. These results suggest that the MNP/QD@MIPs possessed good regeneration ability and stability, and have potential in practical application for sensing of Lyz.

## 4. Conclusions

In this work, a new molecularly imprinted magnetic fluorescent polymer-based biosensor was successfully fabricated and used for direct fluorescence sensing of the target protein Lyz. As an advanced biosensor, the MNP/QD@MIPs combine the pretreatment process and optical detection procedure in one step, which not only facilitates the operation and reduces the cost, but also improves the efficiency and sensitivity of detection in complex matrices. The integration of the magnetic and fluorescence properties of the MNP/QD@MIPs make the whole analysis process simple and fast. The adsorption experiment results showed that the MNP/QD@MIPs sensor has a rapid response and high selectivity and adsorption capacity for Lyz. In addition, the MNP/QD@MIPs successfully employed for fluorescence sensing of Lyz in real samples, and exhibited good stability and recyclability in practical applications. This superior magnetic fluorescence nanocomposites opens up possibilities for the effective analysis and bioseparation of trace proteins in biological samples.

## Figures and Tables

**Table 1:** Detection of lysozyme using MNP/QD@MIPs sensor in real samples.

Sample	Spiked Lysozyme (μM)	Measured (μM)	Recovery (%)	RSD
Egg white 1	0.50	0.51 ± 0.03	101.78	5.29
Egg white 2	1.50	1.51 ± 0.05	97.51	3.15
Urine 1	0.50	0.50 ± 0.02	103.33	3.72
Urine 2	1.50	1.49 ± 0.06	95.40	4.25

**Table 2:** Comparison of the performance of the MNP/QD@MIPs sensor with previously reported MIPs-based sensors for the determination of lysozyme.

Recognition Element	Detection Technique	Linearity (μM)	LOD (μM)	References
MIPs	Electrochemical	0.15–20	0.14	[44]
MIP based on *Naviculasp. frustule*	Fluorescence spectrum	0–1.74	0.10	[48]
MIP@GNR	SERS	5.6 × 10^−2^–2.10	10^−2^	[49]
CDs/SiO_2_/MIP	Fluorescence spectrum	6.9 × 10^−2^–0.69	3.84 × 10^−2^	[50]
QDs embedded MIMs	Fluorescence spectrum	0.1–1.0	1.02 × 10^−2^	[43]
MIP@MNP/QDs	Fluorescence spectrum	0.2–2.0	4.53 × 10^−3^	This work

MIPs: molecular imprinted polymers; GNR: gold nanorods; SERS: surface enhanced Raman scattering; CDs: carbon dots; SPE: solid phase; MIMs: molecular imprinted membranes; LOD: limit of detection.

## Data Availability

The data presented in this study are available upon request from the corresponding author.

## Supplementary Materials

The following are available online at https://www.mdpi.com/article/10.3390/nano11061575/s1. Figure S1. Particle size distribution (a) Mn^2+^: ZnS QDs, (b) MNPs, (c) MNP/QD and (d) MNP/QD@MIPs. Figure S2. XRD of Mn^2+^: ZnS QDs, MNP, and MNP/QDs. Figure S3. EDX of Mn2+: ZnS QDs and MNP/QDs. Figure S4. VSM of MNP. Figure S5. The UV-Vis spectra of MNP, MNP/QDs and MNP/QD@MIPs. Figure S6. The fluorescence spectra of Mn: ZnS QDs. Figure S7. Binding kinetics of MNP/QD@MIPs and MNP/QD @NIPs for lysozyme. Figure S8. Stability and recyclability of MNP/QD@MIPs-based sensor.
