# Supplementary material for: Molecularly Imprinted Magnetic Fluorescent Nanocomposite-Based Sensor for Selective Detection of Lysozyme
Source: Nanomaterials (Basel). 2021 Jun 15;11(6):1575. doi: 10.3390/nano11061575 (PMC8232576; doi:10.3390/nano11061575)
Supplement: Supplementary file 1 [file nanomaterials-11-01575-s001.zip › nanomaterials-1251431-SI.pdf]

Supplementary Materials

Molecularly Imprinted Magnetic Fluorescent Nanocomposite-Based Sensor for Selective Detection of Lysozyme

Xin Zhang [1,*], Bo Tang [2], Yansong Li [1], Chengbin Liu [1], Pengfei Jiao [1] and Yuping Wei [1]

[1] School of Life Science and Agricultural Engineering, Nanyang Normal University, Nanyang 473061, China, li.yansong1989@163.com (Y.L.), liuchengbin@tio.org.cn (C.L.), dongbx@nynu.edu.cn (P.J.), ypwei@ipe.ac.cn (Y.W.)
[2] Key Laboratory of Molecular Medicine and Biotherapy, School of Life Science, Beijing Institute of Technology, Beijing 100081, China, 3120170650@bit.edu.cn
* Correspondence: 21588330@bit.edu.cn

1. Synthesis of *l*-Cysteine Capped-Modified Mn^{2+}:ZnS QDs

l-cysteine-capped ZnS QDs were synthesized according reported methods with some modifications [1]. 1.8 g $ZnSO_4 \cdot 7H_2O$, 0.1 g $MnCl_2 \cdot 4H_2O$, and 0.5 g *l*-cysteine were added into 25 mL deionized water. The mixture was stirred under N_2 and in the dark at 30 °C for 2 h. Then 5 mL of $Na_2S \cdot 9H_2O$ (0.25 mol L^{-1}) was added in dropwise. The above solutions were stirred under N_2 and in the dark for 20 h. The prepared *l*-cysteine-capped modified Mn^{2+}:ZnS QDs were washed with water and ethanol to remove unreacted substances and dried under vacuum.

2. Prepared of Carboxyl-Functionalized Fe_3O_4 MNPs

Carboxyl modified d Fe_3O_4 MNPs were synthesized according reported methods [2]. 3.9 g $FeCl_3 \cdot 6H_2O$, 1.2 g $Na_3Cit_2 \cdot H_2O$, and 1.8 g NaAc were dissolved in 120 mL ethylene glycol and stirred for 30 min. The solution was transferred into a steel autoclave and heated at 200 °C for 12 h. The resultant products were washed and dried under vacuum.

3. Binding Experiments

In all protein rebinding experiments, the MNP/QD@MIPs or MNP/QD@NIPs was dispersed in phosphate buffer with different concentrations of template Lyz at 25 °C. The binding kinetics was measured by detecting the fluorescence intensity changes of MNP/QD@MIPs with added Lyz at different incubation times. Binding isotherm experiments were performed by determining the adsorption capacities of MNP/QD@MIPs and MNP/QD@NIPs for Lyz (0.2 to 2.0 μM). The amount of protein adsorbed (Q, mg/g) by the MNP/QD@MIPs were calculated by:

$$Q = (C_0 - C_e)\frac{V}{W}$$

Where C_0 and C_e (mg mL^{-1}) are the initial concentration and the free concentration of the Lyz or competitive protein at equilibrium, V (mL) is the volume of the initial solution, and W (g) is the weight of the MNP/QD@MIPs or MNP/QD@NIPs.

4. Strategy for Using an MNP-QD@MIPs-Based Sensor for the Detection of Lysozyme

The detection strategy was mainly divided into two steps: (1) the selective separation of lysozyme in samples, and (2) fluorescence detection. (1) The MNP/QD@MIPs was dispersed into the samples. The target lysozyme molecule was specifically bound onto the

MNP/QD@MIPs via the MIP layer. Then MNP/QD@MIPs loaded with lysozyme molecule was magnetically decanted. (2) The collected MNP/QD@MIPs was redispersed in buffer solution, and the same concentration of MNP/QD@MIPs was dispersed in buffer solutions without lysozyme as a control. The fluorescence intensity of each sample was recorded, and the concentration of lysozyme in the samples was calculated. This method did not involve any other pretreatment procedures or additional instruments.

5. Quantum Yields

The quantum yields (QYs) of the MNP/QDs and MNP/QD@MIPs were calculated according to the following equation:

$$\phi_x = \phi_s \lfloor A_s / A_x \rfloor [\ln t_x / \ln t_s][\eta_x / \eta_s]^2$$

Where, *Φ* is the quantum yield, *A* is absorbance at the excitation wavelength, *Int* is the area under the emission peak, and η is the refractive index of the solvent. The subscripts *s* and *x* denote the standard and samples, respectively. Rhodamine B (QY = 69% in ethanol) was used as a standard. The quantum yield (QY) of the MNP/QDs and MNP/QD@MIPs was 23.18% and 18.87 in ethanol.

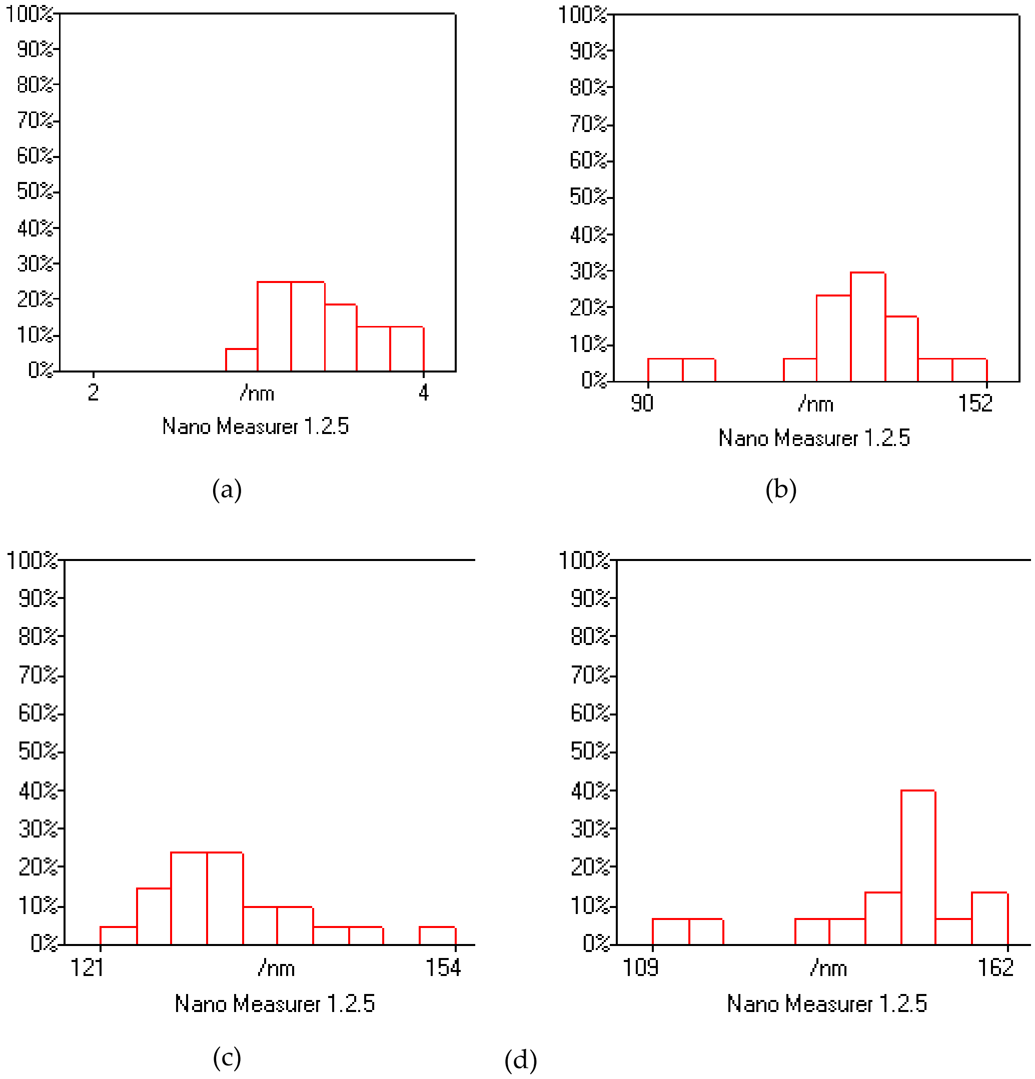

Figure S1. Particle size distribution (**a**) Mn^{2+}:ZnS QDs, (**b**) MNPs, (**c**) MNP/QD and (**d**) MNP/QD@MIPs.

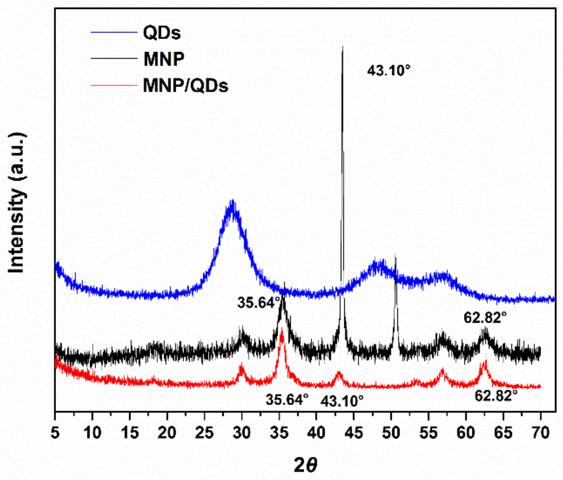

Figure S2. XRD of Mn^{2+}:ZnSQDs, MNP, and MNP/QDs.

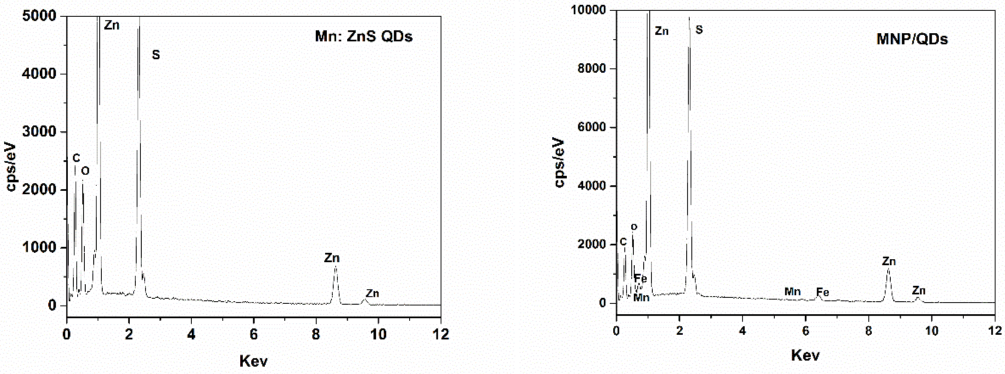

Figure S3. EDX of ZnS QDs and MNP/QDs.

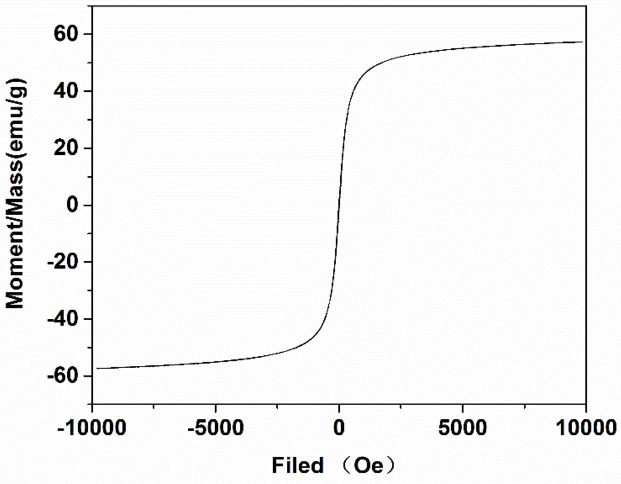

Figure S4. VSM of MNP.

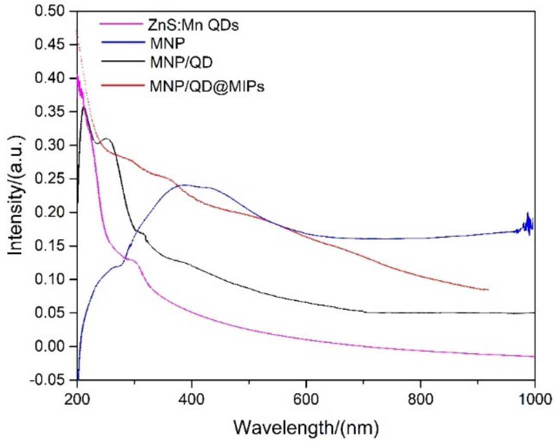

Figure S5. The UV-Vis spectra of MNP, MNP/QDs and MNP/QD@MIPs.

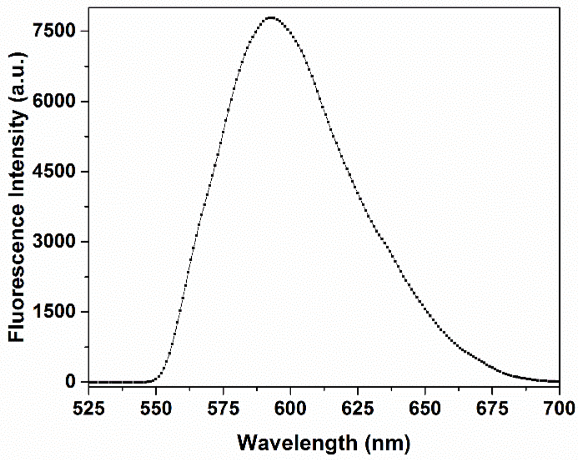

Figure S6. The fluorescence spectra of Mn:ZnS QDs.

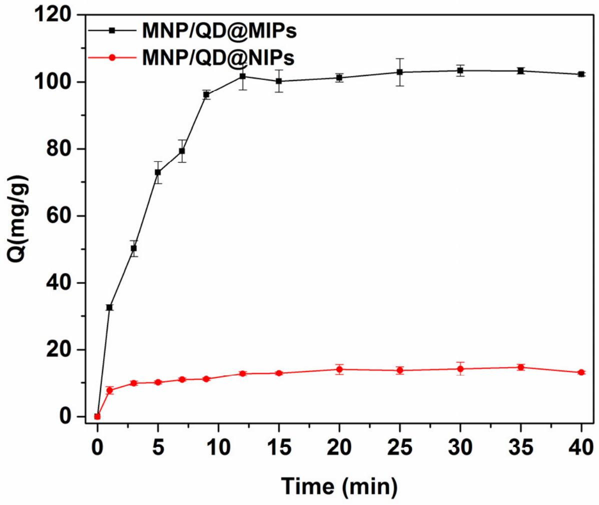

Figure S7. Binding kinetics of MNP/QD@MIPs and MNP/QD @NIPs for lysozyme.

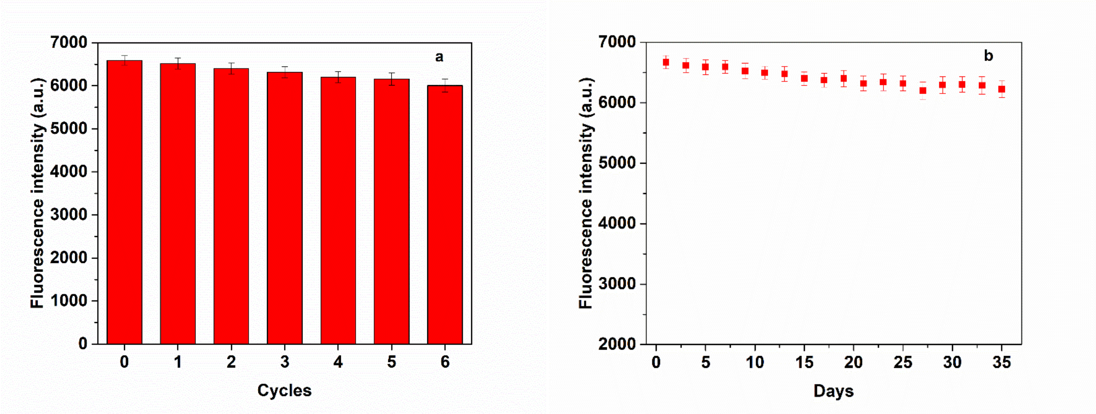

Figure S8. Stability and recyclability of MNP/QD@MIPs based sensor.

Reference

1.Wang, H. F.; He, Y., Ji, T. R.; Yan, X. P. Surface molecular imprinting on Mn-doped ZnS quantum dots for room-temperature phosphorescence optosensing of pentachlorophenol in water *Anal. Chem.* **2009**, *81*:1615-1621.

2.Yang, S.; Zhang, X.; Zhao, W.; Sun, L.; Luo, A. Preparation and evaluation of Fe_3O_4 nanoparticles incorporated molecularly imprinted polymers for protein separation *J. Mater. Sci.* **2016**, *51*, 937–949.
